# Geographic factors influence communities of symbiotic bacterial communities in *Aphis gossypii* across China's major cotton regions

**DOI:** 10.3389/fmicb.2025.1569543

**Published:** 2025-04-01

**Authors:** Abulaiti Alimu, Yu Gao, Jinping Liu, Yanhui Lu

**Affiliations:** ^1^State Key Laboratory for Biology of Plant Diseases and Insect Pests, Institute of Plant Protection, Chinese Academy of Agricultural Sciences, Beijing, China; ^2^Western Agricultural Research Center, Chinese Academy of Agricultural Sciences, Changji, China

**Keywords:** *Aphis gossypii*, bacterial communities, 16S rRNA gene sequencing, environmental factors, cotton

## Abstract

**Introduction:**

Aphids are often infected with diverse bacterial symbionts that enhance their ecological adaptation. While geographic factors significantly influence aphid bacterial communities, research on environmental effects on the cotton aphid *Aphis gossypii* Glover feeding on cotton plants across China's major cotton-growing regions is limited.

**Methods:**

This study examined the influence of geographic factors on the endosymbiotic bacterial community and diversity of *A. gossypii* by analyzing 58 field samples from 24 locations across China's major cotton-growing regions (2021–2022) using 16S rRNA (V3–V4) high-throughput sequencing.

**Results and discussion:**

Our results demonstrate that geography is an important factor in shaping the endosymbiotic bacterial composition and diversity of *A. gossypii*. Among China's three major cotton-growing regions, the Yangtze River Basin exhibited the highest bacterial diversity, followed by the Northwestern Inland Region, and then the Yellow River Basin. *Acinetobacter, Lactobacillus, Serratia*, and *Aeromonas* were more abundant in the Yangtze River Basin, with positive correlations observed for *Acinetobacter, Serratia*, and *Aeromonas* in relation to annual precipitation. In contrast, *Candidatus Uzinur*a, dominant in southern Xinjiang, displayed negative correlations with precipitation and longitude but a positive correlation with altitude, and this report is the first detection of it in *A. gossypii*. *Buchnera* was ubiquitous and negatively associated with both precipitation and temperature, while *Arsenophonus* showed no significant environmental correlations. These findings highlight the distinct influences of geographic factors on *A. gossypii* endosymbiotic communities across China's major cotton-growing regions, broadening our understanding of aphid–endosymbiont–environment interactions and offering potential avenues for biocontrol strategies.

## 1 Introduction

The cotton aphid (*Aphis gossypii* Glover; Hemiptera: Aphididae) is a major global agricultural pest, infesting over 600 plant species and causing substantial economic losses in cotton production through direct feeding and virus transmission (Carletto et al., 2009; Liu et al., 2017; Wu and Guo, 2005). In China, cotton remains one of the most economically important crops, primarily grown in three major regions: the Northwest Inland, the Yellow River Basin, and the Yangtze River Basin. Among these, Xinjiang has emerged as a leading cotton-producing region in China (Feng et al., 2022; Wu and Guo, 2005).

Aphids commonly harbor diverse symbiotic bacteria that play a critical role in their survival, fitness, and ecological adaptability. While, the composition and diversity of symbiotic bacterial communities in aphids are influenced by multiple factors, including genetics, geography, and host plant species (Baumann, 2005; Łukasik et al., 2013; Oliver et al., 2003; Russell and Moran, 2006; Guo et al., 2017; Oliver et al., 2010; Tougeron and Iltis, 2022; Li et al., 2021; Oliver et al., 2005). The symbiotic bacterial communities are quite different among the different aphid species; however, most studies on aphid–bacteria interactions have focused on the pea aphid (*Acyrthosiphon pisum*). In *A. pisum*, host plants play a critical role in shaping bacterial communities, and several previous studies have highlighted significant differences in bacterial community composition and prevalence among populations on different host plants (Ferrari et al., 2012; Gauthier et al., 2015). Geography also exerts a strong influence on these symbiotic associations. For instance, the bacterial community structure of *A.pisum* was strongly influenced by geography (Zhang et al., 2021). Distinct distribution patterns of secondary symbionts—particularly *Regiella insecticola*—were observed in Japanese *A. pisum* populations (Tsuchida et al., 2002). Beyond pea aphids, the grain aphid *Sitobion avenae* harbors symbiotic bacteria with clear geographic distribution patterns, including a strong positive correlation between *Regiella insecticola* infection and environmental parameters such as mean annual temperature and precipitation (Hu et al., 2020). Another study showed that symbiotic bacteria in four cereal aphid species and *A. pisum* in two agroclimatic zones of Chile varied across aphid species, host plants, and geographic regions (Sepúlveda et al., 2017). Moreover, the bacterial communities associated with wheat aphid, *Sitobion miscanthi*, in China's major wheat-growing region affected pesticide resistance and were modulated by environmental factors (Wang et al., 2022).

*Aphis gossypii*, like other aphids, harbors diverse endosymbiotic bacteria that enhance its ecological adaptation. Its bacterial communities are mostly influenced by host plants and geography. For instance, the symbiont diversity in Japanese and Australian populations of *A. gossypii* was primarily shaped by geography rather than host plant (Najar-Rodríguez et al., 2009). Moreover, another study revealed that consistent bacterial community structures within provinces, but found significant inter-province variation in northern China, indicating strong geographical effect (Zhao et al., 2016). In contrast, host plant was a more significant determinant of symbiont composition than geography in several *A. gossypii* populations (Xu et al., 2020). These studies emphasize the complex interplay of factors driving microbial symbiont associations in *A. gossypii*. Despite evidence indicating that geographic factors can shape the bacterial communities of *A. gossypii*, previous research has been based on limited sampling across diverse host plants. Consequently, there has been a comprehensive study of *A. gossypii* populations exclusively associated with cotton in China's three main cotton-producing regions. Here, we performed 16S rRNA (V3–V4) high-throughput sequencing on 58 cotton aphid field samples from 24 cotton fields distributed over these three regions, demonstrating distinct geographic influences on *A. gossypii* symbiotic bacterial communities. These findings expand our understanding of aphid–symbiont–environment interactions and offer new prospects for developing biocontrol strategies.

## 2 Materials and methods

### 2.1 Sampling of *Aphis gossypii* from different geographical regions

**Figure 1:**
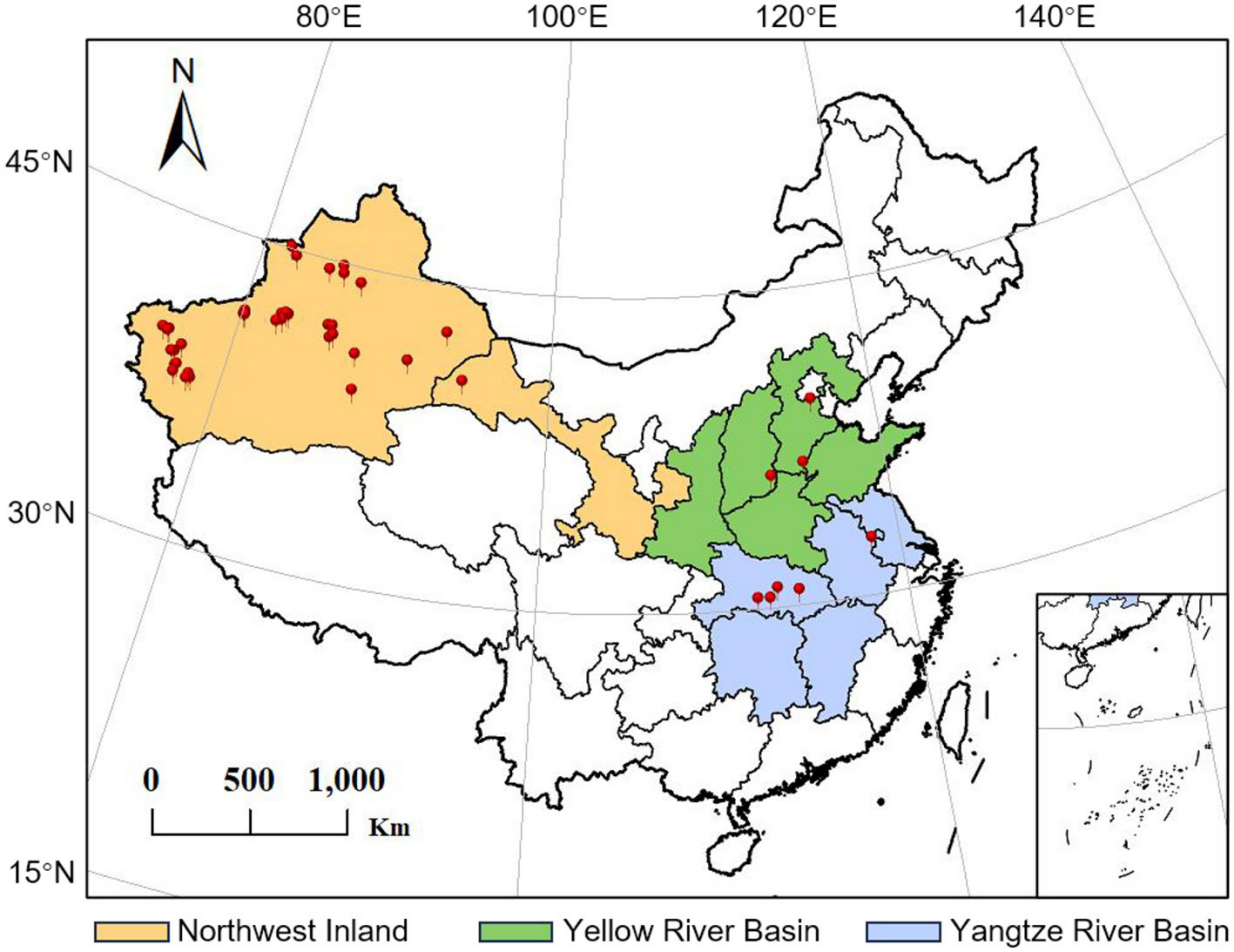
Collections sites of *A. gossypii* from three major cotton-growing regions in China. Detailed information on the specific locations and collection dates is provided in Table 1.

To investigate the diversity of symbiotic microbial communities in different geographical populations of *A. gossypii*, a total of 58 field samples were collected in 2021 and 2022 from 24 different geographic locations across three major cotton-growing regions in China, including the Northwest Inland Cotton Region (Northwest), the Yellow River Basin Cotton Region (Yellow) and the Yangtze River Basin Cotton Region (Yangtze) (Figure 1 and Table 1 for details).

**Table 1:** Sampling information for *A. gossypii* collected from three major cotton-growing regions in China.

**Cotton-growing Area**	**Region**	**ID**	**Long**	**Lati**	**Alti (m)**	**AAP (mm)**	**AAT (°C)**	**Date**
Northwest Inland	Guma, Xinjiang	PS1	78.38	37.56	1,468	34.26	13.53	2,021.06.23
	Guma, Xinjiang	PS2	78.57	37.41	1,435	32.96	13.35	2,021.06.23
	Guma, Xinjiang	PS3	78.33	37.15	1,385	29.41	12.49	2,022.06.25
	Qaghiliq, Xinjiang	YC1	77.46	37.75	1,464	35.81	13.10	2,021.06.23
	Qaghiliq, Xinjiang	YC2	77.52	37.81	1,352	33.88	13.38	2,021.06.23
	Qaghiliq, Xinjiang	YC3	77.46	37.45	1,923	32.01	10.85	2,022.06.24
	Yarkant, Xinjiang	SCH1	77.15	38.37	1,232	52.51	13.79	2,021.06.22
	Yarkant, Xinjiang	SCH2	77.41	38.76	1,187	55.32	13.59	2,021.06.22
	Yarkant, Xinjiang	SCH3	76.95	38.33	1,257	47.96	13.43	2,022.06.24
	Shule, Xinjiang	SL1	76.37	39.27	1,267	87.82	13.05	2,021.06.22
	Shule, Xinjiang	SL2	75.96	39.27	1,289	103.02	12.50	2,021.06.22
	Shule, Xinjiang	SL3	76.32	39.19	1,249	93.87	12.82	2,022.06.25
	Wensu, Xinjiang	WS1	80.42	41.11	1,110	75.98	12.01	2,021.06.23
	Wensu, Xinjiang	WS2	80.47	41.27	1,206	94.01	11.50	2,021.06.23
	Wensu, Xinjiang	WS3	80.53	41.13	1,128	81.34	11.92	2,022.06.27
	Kuqa, Xinjiang	KC1	82.76	41.63	1,019	155.77	12.12	2,021.06.22
	Kuqa, Xinjiang	KC2	83.19	41.71	1,048	160.54	12.01	2,021.06.22
	Kuqa, Xinjiang	KC3	82.99	41.73	1,018	170.53	12.22	2,022.06.27
	Xayar, Xinjiang	SHY1	82.53	41.26	990	123.45	12.73	2,021.06.22
	Xayar, Xinjiang	SHY2	82.84	41.39	993	142.11	12.55	2,021.06.22
	Xayar, Xinjiang	SHY3	82.53	41.26	990	123.45	12.73	2,022.06.27
	Korla, Xinjiang	KEL1	85.79	41.72	900	68.51	12.28	2,021.06.23
	Korla, Xinjiang	KEL2	86.07	41.74	959	62.22	12.22	2,021.06.23
	Korla, Xinjiang	KEL3	85.79	41.72	900	68.51	12.28	2,022.06.26
	Korla, Xinjiang	KEL4	86.07	41.74	959	62.22	12.22	2,022.06.26
	Lopnur, Xinjiang	YL1	86.07	41.15	888	45.55	12.76	2,021.06.21
	Lopnur, Xinjiang	YL2	86.23	41.32	887	48.41	12.51	2,021.06.21
	Lopnur, Xinjiang	YL3	86.07	41.15	888	45.55	12.76	2,022.06.26
	Lopnur, Xinjiang	YL4	86.23	41.32	887	48.41	12.51	2,022.06.26
	Qarkilik, Xinjiang	RQ1	87.84	40.65	849	30.81	11.97	2,021.06.21
	Qarkilik, Xinjiang	RQ2	88.13	38.93	927	29.62	10.58	2,021.06.21
	Qarkilik, Xinjiang	RQ3	87.84	40.65	849	30.81	11.97	2,022.06.25
	Qarkilik, Xinjiang	RQ4	88.13	38.93	927	29.62	10.58	2,022.06.25
	Changji, Xinjiang	CHJ4	87.27	44.02	576	193.56	8.62	2,021.06.21
	Shihezi, Xinjiang	SHZ1	86.01	44.27	473	167.42	9.11	2,021.06.21
	Shihezi, Xinjiang	SHZ2	85.88	44.64	397	155.03	9.23	2,021.06.21
	Shihezi, Xinjiang	SHZ3	86.01	44.27	473	167.42	9.11	2,022.06.26
	Kuytun, Xinjiang	KT1	85.02	44.32	442	192.42	7.43	2,021.06.21
	Kuytun, Xinjiang	KT2	85.02	44.32	442	192.42	7.43	2,022.06.26
	Bole, Xinjiang	BL1	82.62	44.51	526	199.71	10.11	2,021.06.21
	Bole, Xinjiang	BL2	82.16	44.86	452	219.92	8.51	2,021.06.21
	Bole, Xinjiang	BL3	82.16	44.86	452	219.92	8.51	2,022.06.26
	Kumul, Xinjiang	HM1	93.41	42.46	767	29.38	12.02	2,021.06.23
	Kumul, Xinjiang	HM2	96.38	43.64	856	17.43	12.06	2,021.06.23
	Kumul, Xinjiang	HM3	93.41	42.46	767	29.38	12.02	2,022.06.26
	Dunhuang, Gansu	GS1	94.77	40.26	1,142	41.22	10.38	2,022.07.13
Yellow river basin	Langfang, Hebei	LF1	116.36	39.3	23	545.77	13.28	2,021.07.11
	Langfang, Hebei	LF2	116.36	39.3	23	545.77	13.28	2,022.07.16
	Linqing, Shandong	LQ	115.42	36.38	35	582.42	14.30	2,021.08.15
	Xinxiang, Henan	XX2	113.47	35.91	78	718.10	8.63	2,021.07.25
	Xinxiang, Henan	XX1	113.47	35.91	78	718.10	8.63	2,022.08.07
Yangtze river basin	Wuhan, Hubei	WH1	114.33	30.49	31	1,291.63	17.78	2,021.08.23
	Wuhan, Hubei	WH2	114.33	30.49	31	1,291.63	17.78	2,022.08.14
	Qianjiang, Hubei	QJ	112.72	30.23	28	1,121.76	17.50	2,021.08.27
	Jingzhou, Hubei	JZH	112.08	30.25	35	1,088.63	17.37	2,021.08.21
	Tianmen, Hubei	TM	113.16	30.66	32	1,100.49	17.40	2,021.09.03
	Nanjing, Jiangsu	NJ1	118.62	32.41	15	1,033.48	16.18	2,021.08.17
	Nanjing, Jiangsu	NJ2	118.62	32.41	15	1,033.48	16.18	2,022.09.13

In each location, at least one cotton field that had been continuously cultivated with cotton for several years was selected (if a second field was included in the same location, there were > 10 km between fields). Samples of *A. gossypii* were collected from each cotton field using a five-point sampling method, ensuring that the distance between each sampling point was >10 m, and only one cotton plant was selected from each sampling point. We collected 30–50 wingless adult aphids from each sampling point. Samples from the same field were considered as one sample, from which 10 individual aphids were randomly selected from each sampling point, resulting in 50 wingless aphids as the group to be examined. These 50 aphids were placed in a 1.5 mL EP tube containing 1 mL of absolute ethanol and stored at −20°C until examined.

### 2.2 DNA isolation, 16s rRNA gene PCR amplification and high-throughput sequencing

Total genomic DNA was extracted from 58 samples of *A. gossypii* samples (each of 50 aphids) collected from 24 geographical locations. Before DNA extraction, we discarded the ethanol from the sample tubes, and triple-rinsed the aphids with sterile ultrapure water (3 min per rinse). Genomic DNA was extracted from the group of 50 aphids to using the NucleoSpin 96 Tissue DNA kit (Genetic Biotechnology International Trading Co., Shanghai, China) following the manufacturer's protocol. The quality of extracted DNA was assessed by 1.5% agarose gel electrophoresis, while DNA concentration and purity were quantified using a NanoDrop 2000 UV spectrophotometer (Thermo Fisher Scientific, Waltham, MA, USA). DNA samples were subsequently diluted to 100 ng/μL with TE buffer and stored at −20°C.

The V3-V4 hypervariable region of the 16S rRNA gene was amplified from the *A. gossypii* genomic DNA using the universal bacterial primers 338F (5′-ACTCCTACGGGAGGCAGCA-3′) and 806R (5′-GGACTACHVGGGTWTCTAAT-3′) (Yu et al., 2005). Each PCR reaction (20 μL total volume) contained 40–50 ng of template DNA, 0.3 μL of each primer (10 μM), 5 μL of KOD FX Neo buffer, 2 μL of dNTPs (2 mM each), 0.2 μL of KOD FX Neo polymerase, and nuclease-free water to make up the volume to 20 μL. The thermal cycling conditions were as follows: initial denaturation at 95°C for 5 min, 30 cycles of denaturation at 95°C for 30 s, annealing at 50°C for 30 s, and extension at 72°C for 40 s, followed by a final extension at 72°C for 7 min. The completed reaction products were held at 4°C. The PCR products were purified using the Omega DNA Purification Kit (Omega Bio-tek, Norcross, GA, USA) and quantified using a Qsep-400 DNA analyzer (BiOptic, New Taipei City). The purified amplicons were then used for small-fragment library construction. Paired-end sequencing (2 × 250 bp) was performed on an Illumina NovaSeq 6000 platform (Beijing Biomics Technology Co., Ltd., Beijing, China).

### 2.3 Bioinformatic analysis

The paired-end reads were assembled using FLASH (version 1.2.11) (Magoč and Salzberg, 2011) with a minimum overlap of 10 bp and a maximum mismatch ratio of 20%, producing raw tags. These raw tags were then utilized for operational taxonomic unit (OTU) clustering. Tags with an average quality score below 20 or a length shorter than 75% of their original size were filtered using Trimmomatic (version 0.33) (Bolger et al., 2014). Primer sequences were identified and removed with Cutadapt (version 1.8.3) (Kechin et al., 2017). Chimeric sequences were removed using UCHIME (Edgar et al., 2011) by comparing query fragments to a reference database, producing clean tags. The clean tags were clustered into OTUs at a 97% similarity threshold using USEARCH (version 10.0) (Edgar, 2013) and OTUs representing < 0.005% of the total sequence count were filtered out (Bokulich et al., 2013). Taxonomic annotation of the filtered OTUs was carried out using QIIME2 (Bolyen et al., 2019), applying a Naive Bayes classifier trained on the SILVA database (version 138.1) (Quast et al., 2013) at a confidence threshold of 70%. Both alpha diversity and beta diversity metrics were calculated using QIIME2, and visualized using the ggplot2 package (Ginestet, 2011) Redundancy analysis (RDA) was performed in R using vegan package (Oksanen et al., 2018) to evaluate environmental factors influencing microbial community composition. All analyses were conducted on the BMKCloud platform.

### 2.4 Environmental factor data collection

Th0065 sampling locations varied in altitude, precipitation, temperature, and latitude/longitude. In this study, the historical monthly precipitation and temperature data (2010–2021) were downloaded from the WorldClim website (https://worldclim.org/data/monthlywth.html). Latitude and altitude were recorded during sampling using a mobile phone's built-in compass app. Precipitation and temperature data were extracted from the WorldClim dataset (Harris et al., 2020) in ArcMap 10.8 based on the recorded coordinates.

### 2.5 Statistical analysis

For the downstream statistical analysis, samples were categorized into three geographical groups: Northwest (46 samples from 16 locations), Yellow (5 samples from 3 locations), and Yangtze (7 samples from 5 locations).

Alpha diversity indices (Simpson, Shannon, Chao1, and ACE) were calculated to assess bacterial community diversity and richness across the three geographical groups using the R package vegan. Pairwise inter-group significance was calculated using the non-parametric Wilcoxon test (*P* < 0.05), and the results were visualized using the R package ggplot2. Principal coordinate analysis (PCoA) based on the Weighted UniFrac distances matrix at the OTU level was conducted using the R package GuniFrac (Chen et al., 2012), and the results were visualized with the R package ggplot2. To further assess the significance of differences in bacterial communities across the geographical regions, PERMANOVA and ANOSIM analysis values at a 95% confidence level were calculated using the anoism and adonis functions of in the vegan package.

The influence of environmental factors (precipitation, temperature, altitude, and geographic coordinates) on microbial community composition were assessed using redundancy analysis (RDA) and Mantel tests at the genus level using the vegan package. Pearson correlation analysis was conducted to explore the relationships between environmental factors and the relative abundances of the 11 common endosymbionts. Significant correlations were identified using a correlation coefficient threshold of 0.5 and a significance level of *P* < 0.05. Correlation heatmaps were generated using the pheatmap package in R.

## 3 Results

### 3.1 Bacterial communities in *Aphis gossypii* from three different geographical regions

High-throughput sequencing of 58 samples generated a total of 4,398,796 paired-end reads. After quality control and assembly, 4,335,095 clean reads were obtained, with a minimum of 42,118 clean reads and an average of 74,743 clean reads per sample. These reads were clustered into 42,757 OTUs and classified into 51 phyla, 130 classes, 403 orders, 886 families, 2,168 genera, and 3,084 species.

At the phylum level, *Proteobacteria* was the dominant one across all regions, though its relative abundance varied substantially: 89.7% in the Northwest Inland, 95.7% in the Yellow River Basin, and 42.8% in the Yangtze River Basin. *Firmicutes* was the second most abundant phylum in the Yangtze region (14.2%), while its abundance was notably lower in the Northwest Inland (3.8%) and Yellow River Basin (2.1%) regions. In addition, the phyla *Bacteroidota* and *Actinobacteriota* exhibited substantially higher abundance in the Yangtze region, at 8.5 and 6.6%, respectively, compared to 3.5% and 0.8% in the Northwest, and 0.6% and 0.4%. in the Yellow River Basin (Supplementary Table 1).

**Figure 2:**
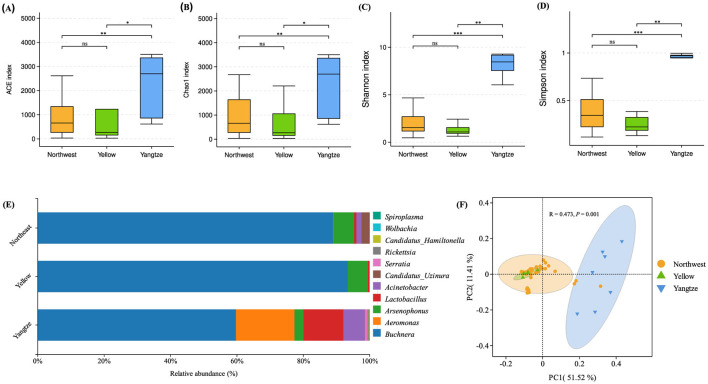
Bacterial diversity in *A. gossypii* across cotton-growing regions (Northwest Inland, Yellow River Basin, and Yangtze River Basin) analyzed via 16S rRNA sequencing. Bacterial community richness and diversity were evaluated using the ACE **(A)**, Chao1 **(B)**, Shannon **(C)**, and Simpson **(D)** indices. Significant differences among groups were determined using the Wilcoxon test at the OTU level. The relative abundances of symbiotic bacteria at the genus level are depicted in panel **(E)**, while principal component analysis (PCoA) based on Weighted UniFrac distances at the OTU level is presented in panel **(F)**. Significance levels: *P < 0.05, **0.001 < *P* < 0.05, ***P < 0.001. Not significant (ns): *P* ≥ 0.05

At the genus level, the primary symbiont *Buchnera* was detected alongside other commonly encountered endosymbiotic genera, including *Arsenophonus, Candidatus Hamiltonella, Wolbachia, Rickettsia, Serratia, Spiroplasma, Candidatus Uzinura, Acinetobacter, Aeromonas*, and *Lactobacillus* (Figure 2E; Table 2). *Buchnera* predominated in all three regions, reaching its highest relative abundance in the Yellow River Basin (93.4%), followed by the Northwest Inland (89.0%) and the Yangtze River Basin (59.7%). *Arsenophonus* showed moderate levels in the Northwest Inland (6.3%) and the Yellow River Basin (6.1%), but declined to 2.7% in the Yangtze River Basin. *Candidatus Uzinura* was generally low, detected at 2.4% in the Northwest Inland but almost absent in the Yellow River Basin (0.01%) and the Yangtze River Basin (0.06%). By contrast, *Aeromonas, Lactobacillus*, and *Acinetobacter* reached higher abundances in the Yangtze River Basin (17.6, 12.0, and 6.4%, respectively), while remaining markedly lower in the Northwest Inland (0.06, 0.65, and 1.56%), and effectively undetected in the Yellow River Basin except for *Lactobacillus* (0.39%) and *Acinetobacter* (0.06%). *Serratia* and *Rickettsia* occurred at low levels across all regions, with *Serratia* slightly more abundant in the Yangtze River Basin (0.84%) compared to the Northwest Inland (0.01%) and the Yellow River Basin (0.004%). *Rickettsia* similarly peaked in the Yangtze River Basin (0.35%) relative to the Northwest Inland (0.01%) and the Yellow River Basin (0.07%). Other genera, including *Candidatus Hamiltonella, Wolbachia*, and *Spiroplasma*, were consistently present at very low abundances. *Candidatus Hamiltonella* remained at 0.01% in both the Northwest Inland and Yellow River Basin, increasing to 0.14% in the Yangtze River Basin. *Wolbachia* followed a similar trend, peaking at 0.13% in the Yangtze River Basin compared to 0.02% in the Northwest Inland and 0.01% in the Yellow River Basin. *Spiroplasma* appeared only in trace amounts (0.03% in the Northwest Inland and 0.02% in the Yangtze River Basin) and was absent in the Yellow River Basin.

**Table 2:** Relative abundances of symbionts in *A. gossypii* from three major cotton-growing regions in China.

**Genus**	**Northwest**	**Yellow**	**Yangtze**
*Buchnera*	88.9%	93.4%	59.7%
*Aeromonas*	0.06%	0.00%	17.6%
*Arsenophonus*	6.3%	6.1%	2.7%
*Lactobacillus*	0.7%	0.4%	12.0%
*Acinetobacter*	1.6%	0.06%	6.4%
*Candidatus_Uzinura*	2.4%	0.01%	0.06%
*Serratia*	0.01%	0.00%	0.84%
*Rickettsia*	0.01%	0.07%	0.35%
*Candidatus_Hamiltonella*	0.01%	0.01%	0.14%
*Wolbachia*	0.02%	0.01%	0.13%
*Spiroplasma*	0.03%	0.00%	0.02%

### 3.2 Bacteria diversity in *Aphis gossypii* from three different geographical regions

When we assessed alpha diversity indices (ACE, Chao1, Simpson, and Shannon) and phylogenetic diversity (PD) across the three regions, we found significant variation (Wilcoxon test, *P* < 0.05) (Figures 2A–D; Table 3). Samples from the Yangtze region exhibited notably higher diversity in all metrics (PD: mean = 217.77, SE = 49.91, Shannon: mean = 8.26, SE = 0.43, Simpson: mean = 0.97, SE = 0.01, ACE: mean = 2,167.72, SE = 490.47, Chao1: mean = 2,166.14, SE = 489.91). In contrast, the Northwest Inland region showed moderate diversity (PD: mean = 109.47, SE = 9.39, Shannon: mean = 2.60, SE = 0.27, Simpson: mean = 0.39, SE = 0.03, ACE: mean = 808.53, SE = 95.48, Chao1: mean = 898.05, SE = 111.18), while the Yellow River Basin exhibited the lowest overall values (PD: mean = 74.76, SE = 28.88, Shannon: mean = 1.29, SE = 0.30, Simpson: mean = 0.25, SE = 0.04, ACE: mean = 813.68, SE = 531.56, Chao1: mean = 674.92, SE = 397.78). Statistical analysis using the Wilcoxon test confirmed significant differences in alpha diversity indices among the regions, with the Yangtze region showing highly significant differences in indices compared to both the Northwest Inland (Simpson: *P* < 0.001; Shannon: *P* < 0.001; ACE: *P* < 0.01; Chao1: *P* < 0.01) and Yellow River Basin (Simpson: *P* < 0.01; Shannon: *P* < 0.01; ACE: *P* < 0.05; Chao1: *P* < 0.05). In contrast, no significant differences were found in the four alpha diversity indices between the Northwest and Yellow River regions (*P* > 0.05 for all indices).

**Table 3:** The alpha diversity indices (mean, SE) (ACE, Chao1, Simpson, and Shannon) and phylogenetic diversity (PD) of microbial symbionts of *A. gossypii* from three major cotton-growing regions in China.

**Region**	**PD**	**ACE**	**Chao1**	**Simpson**	**Shannon**
Northwest	109.47 ± 9.39	808.53 ± 95.48	898.05 ± 111.18	0.39 ± 0.03	2.60 ± 0.27
Yellow	74.76 ± 28.88	813.68 ± 531.56	674.92 ± 397.78	0.25 ± 0.04	1.29 ± 0.30
Yangtze	217.77 ± 49.91	2,167.72 ± 490.47	2,166.14 ± 489.91	0.97 ± 0.01	8.26 ± 0.43

Beta diversity analysis using Weighted UniFrac-based principal coordinate analysis (PCoA) revealed distinct clustering of bacterial communities among the three geographic regions (Figure 2F). Notably, samples from the Yangtze region formed a clearly separate cluster, reflecting significant differences in community structure relative to those from the Yellow River Basin or Northwest Inland (PERMANOVA: *R*^2^ = 0.166, *P* = 0.001; ANOSIM: *R* = 0.321, *P* = 0.009).

These results indicate that the Yangtze region has greater richness and abundance in the symbiotic bacterial community of *A. gossypii* than the other two regions.

### 3.3 The effect of different environmental conditions on structuring of symbiont communities in *Aphis gossypii*

Environmental factors significantly influenced the distribution and composition of symbiotic bacterial communities in *A. gossypii* across from the three geographical regions (Northwest Inland, Yangtze River Basin, and Yellow River Basin). Mantel test results (Table 4) identified annual average precipitation (AAP, *r* = 0.7113, *P* = 0.001) as the most significant environmental factor, followed by latitude (Lati, *r* = 0.6733, *P* = 0.001), annual average temperature (AAT, *r* = 0.5821, *P* = 0.001), longitude (Long, *r* = 0.4393, *P* = 0.001), and altitude (Alti, *r* = 0.3164, *P* = 0.001).

**Table 4:** Mantel test results between environmental factors and bacterial communities in *A. gossypii* from three major cotton-growing regions in China.

**Environmental factors**	**Mantel_test_r_statistic**	***p*-value**
AAP	0.71131573	0.001
Lati	0.673339816	0.001
AAT	0.58207047	0.001
Long	0.439287325	0.001
Alti	0.31641519	0.001

**Figure 3:**
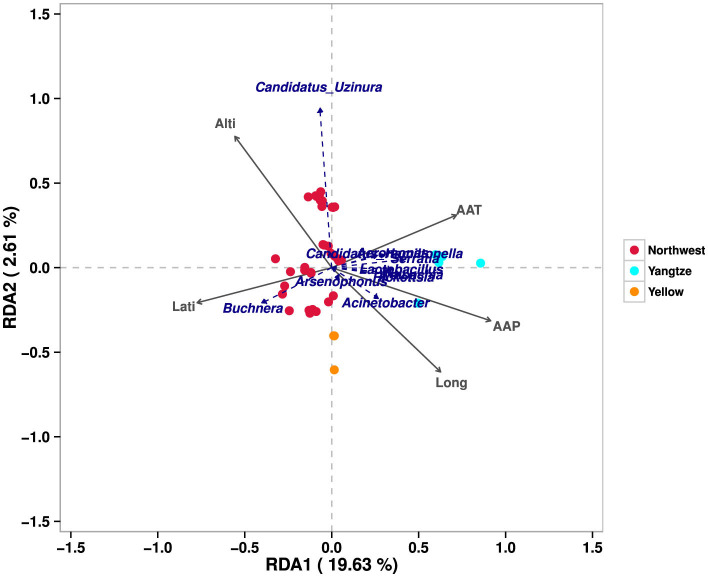
Redundancy analysis (RDA) bacterial communities and environmental factors. This figure illustrates the relationship between symbiotic bacterial communities in *A. gossypii* and various environmental factors across three major cotton-growing regions in China. Each colored point represents populations of *A. gossypii* from distinct geographical regions: red represents the Northwest Inland, cyan denotes the Yangtze River Basin, and orange corresponds to the Yellow River Basin. The blue text highlights the different symbiotic bacterial taxa identified within these populations. Additionally, the arrows signify the environmental factors; an angle of < 90° between two arrows indicates a positive correlation between the corresponding factors.

These findings indicate that the bacterial communities associated with *A. gossypii* are significantly influenced by geographic factors. The redundancy analysis (RDA) results (Figure 3) further confirmed that environmental conditions play a important role in shaping cotton aphid‘s symbiotic bacterial communities, emphasizing the complex interplay between ecological variables and microbial diversity across different geographic contexts.

**Figure 4:**
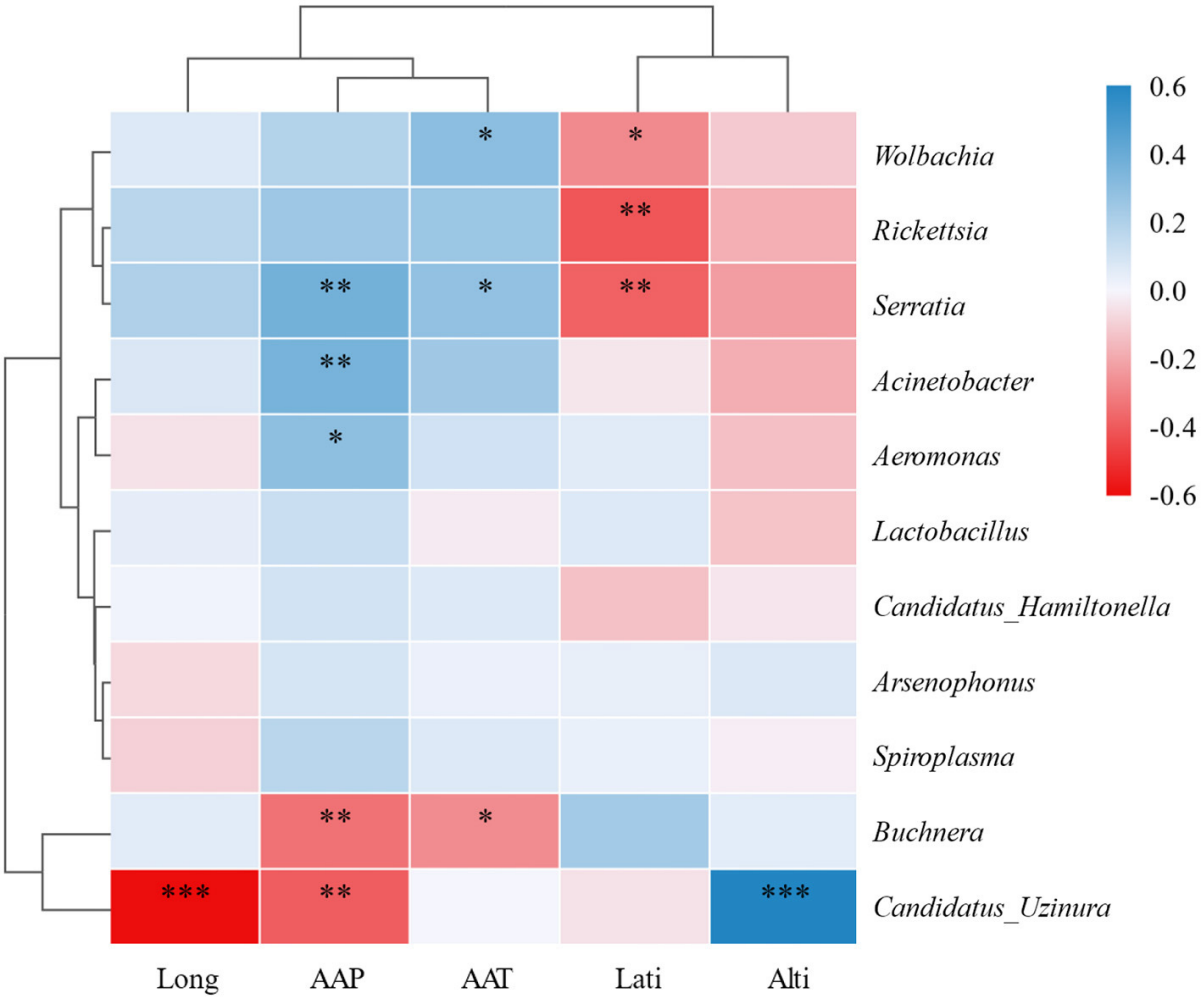
Heatmap of correlations between symbiotic bacteria in *Aphis gossypii* and environmental factors. Rows represent environmental factors, and columns represent symbiotic bacteria. Color intensity reflects correlation strength and direction: darker blue indicates stronger negative correlations, darker red indicates stronger positive correlations. Significance levels: *P < 0.05, **0.001 < *P* < 0.05, ***P < 0.001. Pearson correlation was used with a coefficient threshold of 0.1 and significance set at *P* < 0.05.

Additionally, the heatmap based on Pearson correlation coefficients (threshold = 0.1, significance *P* < 0.05) corroborated the associations identified in the Mantel test and RDA. Notably, *Candidatus Uzinura* exhibited significant negative correlations with longitude (*r* < −0.6, *P* < 0.001) and AAP (*r* < −0.5, *P* < 0.01), while showing a strong positive correlation with altitude (r > 0.6, *P* < 0.001). Conversely, *Serratia* displayed a significant negative correlation with AAP (*r* < −0.6, *P* < 0.01), suggesting its lower occurrence in regions with high precipitation. Furthermore, *Acinetobacter* demonstrated positive correlations with both AAP (*r* > 0.4, *P* < 0.01) and AAT (*r* > 0.4, *P* < 0.01) (Figure 4). These correlations enhance our understanding of how specific environmental factors shape the composition of symbiotic bacterial communities in *A. gossypii*.

## 4 Discussion

We analyzed 58 field samples from 24 locations across China‘s three major cotton-growing regions during 2021–2022 using 16S rRNA (V3–V4) sequencing. Our results indicate that geographic factors, such as precipitation, temperature, altitude, longitude, and latitude, significantly influenced the diversity and composition of bacterial communities in *A. gossypii*. Previous studies have similarly highlighted the important role of environmental factors in shaping aphid endosymbiont communities in various geographic regions (Najar-Rodríguez et al., 2009; Zhao et al., 2016; Martins et al., 2023; Sepúlveda et al., 2017; Tsuchida et al., 2002). In contrast, some research suggests that host plants may have a stronger influence than geography on symbiotic bacteria composition in *A. gossypii* populations (Xu et al., 2020). However, because all *A. gossypii* samples in our study were collected exclusively from cotton plants, host plant variability likely had minimal impact on our results. Their (Xu et al., 2020) study sampled *A. gossypii* from 25 different host plant families, which may account for the differing conclusions regarding host plant influence on symbiont composition. Moreover, another study indicated that geographic factors had a greater effect on the diversity of symbiotic bacteria in *A. gossypii* than host plants (Najar-Rodríguez et al., 2009). These studies highlight the complex interplay among geography, host plants, and the community structure of symbiotic bacteria in *A. gossypii*, warranting further investigation. Our results contribute to this discussion and highlight the importance of understanding these dynamics to elucidate the factors driving microbial community structures in significant agricultural pests.

In this study, we detected *Buchnera*, an obligate endosymbiont of aphids, in all samples (Supplementary Figure 1), confirming its crucial role in aphid physiology, particularly in amino acid synthesis and nutrient provision (Brinza et al., 2009; Douglas, 1998). Heatmap analysis revealed a negative correlation between *Buchnera* abundance and precipitation and temperature (*P* < 0.05). Despite its lower relative abundance in the Yangtze River Basin (59.7%) compared to the Northwest inland (88.9%) and Yellow River Basin (93.4%), it significantly surpassed other symbionts (Figure 3; Table 2). *Arsenophonus*, which was consistently identified as the predominant facultative endosymbiont in *A. gossypii*, showed the highest infection prevalence and relative abundance among the symbiotic bacteria analyzed, aligning with previous studies (Xu et al., 2020; Zhang et al., 2021; Zhao et al., 2016), while *Arsenophonus* exhibited no significant correlation with environmental factors. Furthermore, a recent study indicated a significant correlation between gossypol concentrations and *Arsenophonus* abundance, suggesting that this symbiont confers adaptive advantages to *A. gossypii* in gossypol-rich environments (Chang et al., 2022). Given that all *A. gossypii* samples in our research were sourced from cotton plants, known for their high gossypol content, this likely accounts for the observed high prevalence and relative abundance of *Arsenophonus* in our results.

Moreover, *Wolbachia* was positively correlated with temperature and precipitation but was negatively associated with latitude. A recent study reported positive correlations between *Wolbachia* and both precipitation and temperature (Yang et al., 2023). *Rickettsia* also showed a negative correlation with latitude, while *Serratia* was negatively correlated with latitude and had a weak but positive association with temperature and precipitation. In contrast, *Acinetobacter* and *Aeromonas* were positively correlated with precipitation and weakly but negatively correlated with latitude (Figure 3). In contrast, *Aeromonas*, a facultative symbiont commonly associated with aquatic or semi-aquatic insects (Dubey et al., 2022), was predominantly enriched in the Yangtze River Basin, a humid region characterized by high rainfall. In contrast, *Candidatus Uzinura* exhibited a significant positive correlation with altitude and negative correlations with both precipitation and longitude. It was predominantly detected in cotton aphids from southern Xinjiang (Guma, Qaghiliq, Yarkant, Shule, Wensu, Kuqa, and Qarkilik), where its relative abundance consistently exceeded 2% (Supplementary Figure 2). This region features fruit-cotton intercropping systems, such as jujube-cotton cultivation, which create favorable microhabitats for armored scale insects, significant agricultural pests of fruit and nut trees (Wang et al., 2021; Wei et al., 2016). As a primary endosymbiont of armored scales, *Candidatus Uzinura* plays a critical role in nutritional supplementation (Gruwell et al., 2012, 2007). These systems likely facilitate the symbiont's distribution and proliferation, leading to selective pressures that promote horizontal transmission to other arthropods, including cotton aphids. While direct evidence of *Candidatus Uzinura* in *A. gossypii*, or its horizontal transfer from armored scale insects, is lacking, horizontal symbiont transmission among arthropods has been documented (Chrostek et al., 2017; Gonella et al., 2015). Furthermore, this microbe‘s detection in southern Xinjiang, near the arid Taklamakan Desert, aligns with findings of this endosymbiont in insects from extremely dry environments (Hakobyan et al., 2023). These observations suggest that *Candidatus Uzinura* may confer drought resilience to its host insects. Further research is essential to elucidate its role in host survival, ecological significance, and mechanisms of horizontal transmission across arthropod species.

We investigated the endosymbiotic bacterial communities and diversity of *Aphis gossypii* across China's three major cotton-growing regions. Our findings indicate that geographic factors exert the most significant influence on the composition and diversity of the symbiont community in *A. gossypii*. Additionally, this study highlights the critical relationships between symbiont communities and environmental factors—including precipitation, temperature, altitude, longitude, and latitude—that may have been underestimated in prior research. Furthermore, this study broaden our understanding of aphid–endosymbiont–environment interactions and offering potential avenues for biocontrol strategies.

## Data Availability

The raw sequence data reported in this paper have been deposited in the Genome Sequence Archive (Chen et al., 2021) at the National Genomics Data Center (CNCB-NGDC Members and Partners., 2022), China National Center for Bioinformation / Beijing Institute of Genomics, Chinese Academy of Sciences (GSA: CRA024049) and are publicly accessible at https://ngdc.cncb.ac.cn/gsa/, project number PRJCA037416.
