# Supplementary material for: Geographic factors influence communities of symbiotic bacterial communities in Aphis gossypii across China's major cotton regions
Source: Front Microbiol. 2025 Apr 1;16:1569543. doi: 10.3389/fmicb.2025.1569543 (PMC11998284; doi:10.3389/fmicb.2025.1569543)
Supplement: Supplementary file 1 [file Data_Sheet_1.docx]

**Supplementary information**

TABLE S1 Relative abundance of microbial communities associated with *A. gossypii* from three major cotton-growing regions in China

| Phylum | Northwest | Yellow | Yangtze |
| --- | --- | --- | --- |
| *Proteobacteria* | 89.7% | 95.7% | 42.8% |
| *Firmicutes* | 3.9% | 2.1% | 14.2% |
| *Bacteroidota* | 3.5% | 0.6% | 8.5% |
| *Actinobacteriota* | 0.8% | 0.4% | 6.6% |
| *Cyanobacteria* | 0.1% | 0.06% | 6.5% |
| *Acidobacteriota* | 0.6% | 0.3% | 5.3% |
| *Chloroflexi* | 0.2% | 0.2% | 3.6% |
| *unclassified_Bacteria* | 0.5% | 0.1% | 3.2% |
| *Gemmatimonadota* | 0.1% | 0.06% | 2.0% |
| *Fusobacteriota* | 0.04% | 0.02% | 1.8% |
| *Planctomycetota* | 0.07% | 0.03% | 1.5% |
| *Verrucomicrobiota* | 0.15% | 0.13% | 0.9% |
| *Myxococceota* | 0.07% | 0.03% | 0.90% |
| *Patescibacteria* | 0.09% | 0.04% | 0.46% |
| *Desulfobacterota* | 0.09% | 0.05% | 0.39% |
| Others | 0.2% | 0.12% | 1.5% |
| Unknown | 0.00% | 0.00% | 0.02% |

FIGURE S1 Bar plots of microbial communities associated with *A. gossypii* from three major cotton-growing regions in China
